# Long-Term Follow-Up After Successful Trabeculectomy: A Case Report of Reversal of Cupping and Recovery of Visual Field Progression

**DOI:** 10.7759/cureus.13520

**Published:** 2021-02-23

**Authors:** Shunsuke Nakakura, Ryo Asaoka, Yoshiaki Kiuchi

**Affiliations:** 1 Ophthalmology, Saneikai Tsukazaki Hospital, Himeji, JPN; 2 Ophthalmology, Seirei Hamamatsu General Hospital, Hamamatsu, JPN; 3 Ophthalmology, Seirei Christopher University, Hamamatsu, JPN; 4 Ophthalmology, Hiroshima University, Hiroshima, JPN

**Keywords:** glaucoma, trabeculectomy, reversal of cupping, visual field recovery, adult

## Abstract

Glaucoma is one of the leading causes of blindness worldwide, and reduction of intraocular pressure (IOP) is the only available evidence-based treatment that reduces visual field deterioration in glaucoma. We present a representative case of structural recovery and recovery of visual field progression after successful trabeculectomy (TLE) with long-term follow-up. A 35-year-old woman with glaucoma visited our hospital in 2008. The IOP in her right eye was 11 mmHg at the first visit, and subsequently increased to values in the high teens to 20 mmHg despite treatment with anti-glaucoma eye drops. During this period, the progression of this eye’s visual field deterioration was fast (mean deviation [MD] slope = −0.63 dB/year) and the optic disc cupping was advanced. In the seven-year period after successful TLE in 2014, the IOP declined to between 8 and 12 mmHg without any anti-glaucoma medication. During the first two years after TLE, the MD values were poorer than those before TLE. However, by 2020, MD values improved gradually to a state better than that before the surgery (MD slope during this period was +0.75 dB/year). The appearance of the optic disc was monitored using fundus photography, which showed optic disc morphological changes during topical glaucoma medication before and after TLE. In particular, a remarkable reversal of optic disc cupping enlargement started at two weeks after TLE, and the optic disc shape in 2021 was similar to that in 2008.

Minimally invasive glaucoma surgeries are often preferred; however, our findings suggest that successful TLE followed by long-term rigorous IOP control can result in structural recoveries. Additionally, despite the deterioration in visual field in the early years after TLE, in the long term, it may settle down to the same level before the surgery with recovery of visual field progression, which may be a part of functional recovery.

## Introduction

Trabeculectomy (TLE) is an important surgical procedure for glaucoma because it can be applied to all glaucoma types and it can lower the intraocular pressure (IOP) to a higher degree than that achieved by recently developed minimally invasive glaucoma surgeries [1-4]. However, complications associated with TLE, such as loss of visual acuity, hypotony, infection, cataracts, bleb leakage, and cataract progression, among others, have discouraged some surgeons from practicing this procedure [5]. Nevertheless, TLE surgery may benefit patients by decreasing the depth of the lamina cribrosa (reversal of cupping) and increasing the minimum rim width and area of the optic disc [6-10]. Additionally, TLE may slow the rate of perimetric decay from glaucoma and improve the long-term cell function of the retinal ganglion cells [11]. However, evidence for these benefits is limited due to lack of reporting during long-term follow-ups on morphological and visual field recoveries following successful TLE [12,13].

Here, we present a case describing 12 years of follow-up (six years both before and after TLE) on a patient whose visual field sustained remarkably with the recovery of visual field progression, along with a reversal of glaucomatous optic disc changes after TLE. Although minimally invasive glaucoma surgeries are often preferred [1-4], this case report suggests that sustained maintenance of low IOP after TLE is important.

## Case presentation

**Figure 1:**
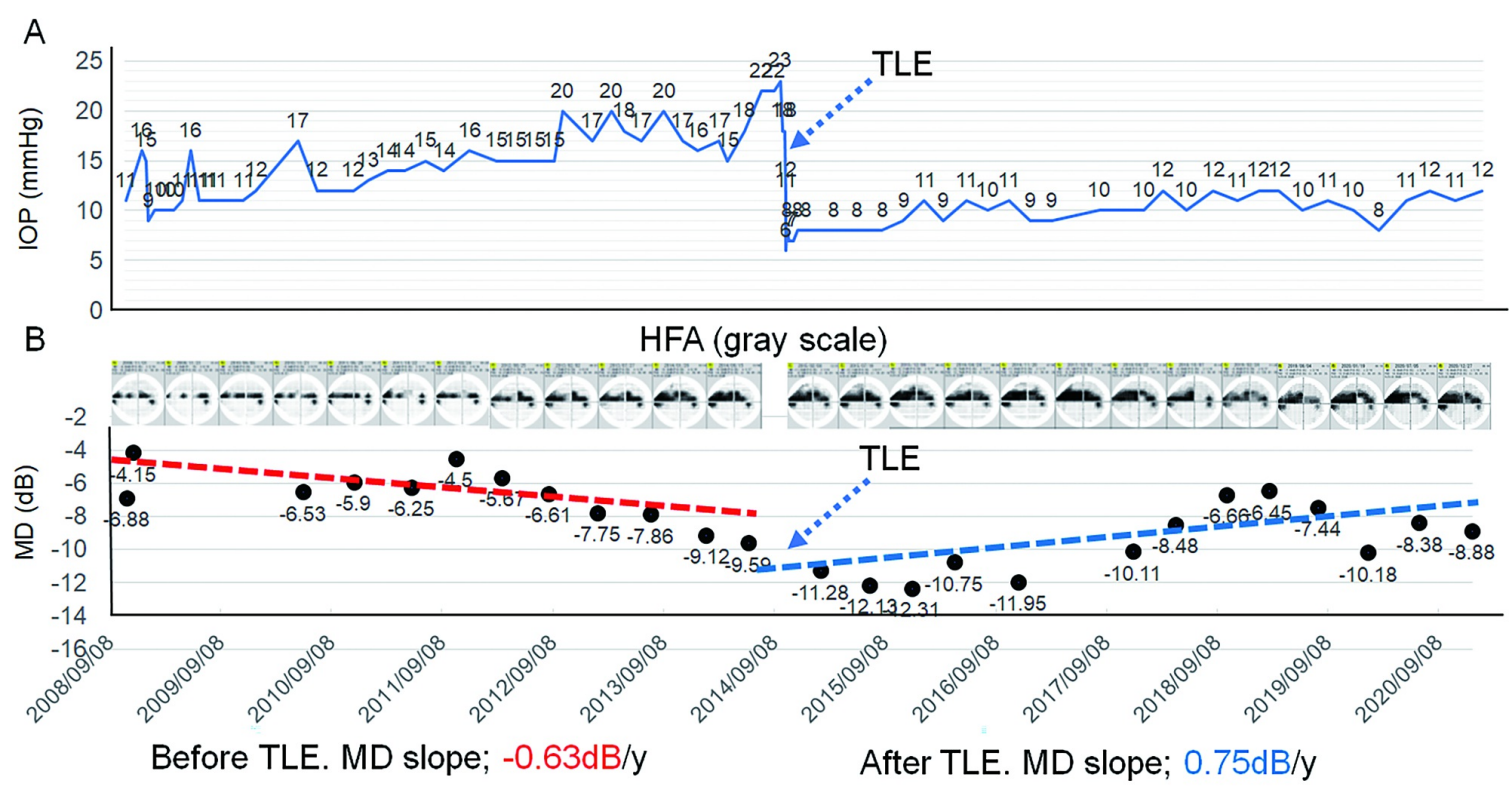
Progress of visual field and intraocular pressure during 12 years (A) IOP measured by Goldmann applanation tonometry. (B) MD and gray scale measured by the Humphrey visual field analyzer (HFA). IOP, intraocular pressure; MD, mean deviation; TLE, trabeculectomy. Dashed arrow indicates the timing of TLE.

This report received approval from the Institutional Review Board of Saneikai Tsukazaki Hospital (IRB no. 201061) and was performed according to the tenets of the Declaration of Helsinki. In 2008, a 35-year-old female patient at Tsukazaki Hospital was diagnosed with primary open-angle glaucoma in the right eye (the left eye was normal). Her right and left eye IOPs were 11 and 15 mmHg, respectively. The visual acuity values in her right and left eyes were 0.4 (1.5 × S − 0.75D = C − 0.5DAx170°) and 1.5 (1.5), respectively. Right and left central corneal thickness values were 448 and 461 μm, respectively. The axial length was 24.4 mm in both eyes. The optic disc of the right eye appeared glaucomatous, with inferior rim thinning and defects in the retinal nerve fiber layer (vertical cup/disc [C/D] ratio = 0.72). The mean deviation (MD) as measured by the Humphrey visual field analyzer (Carl Zeiss Inc., Dublin, CA; Swedish Interactive Threshold Algorithm [SITA]-standard, 24-2) was −6.88 dB in the right eye. Consequently, we started latanoprost 0.005% in the right eye. During three years of this treatment, however, the IOP continued to increase to 14-16 mmHg, whereas the MD value gradually decreased. Considering the thinness of her central cornea, we added a fixed combination of dorzolamide 1% and timolol 0.5% in the right eye in 2011. Nevertheless, the IOP increased to between 17 and 20 mmHg during 2012. Thus, we performed a 360-degree selective laser trabeculoplasty in 2013 (top panel, Figure 1).

The same eye drop regimen as that before the operation was administered; however, the IOP did not decrease, and reached 22 mmHg in June 2014 (Figure 1A). Thereafter, we performed TLE with 0.04% mitomycin C. Before the surgery, right eye MD was −9.59 dB and the MD slope was −0.63 dB/year (Figure 1B). After the surgery, IOP values ranged from 8 to 12 mmHg, without any medication, until 2021. Once MD deteriorated during the two years after TLE, the visual field progression began recovering and subsequently reverted to the pre-TLE level. The post-surgery MD slope was 0.75 dB/year between September 2014 and December 2020 and the MD at the final visit was −8.66 dB, similar to previous TLE. Moreover, the visual filed was sustained by reduction in the IOP efficiency caused by successful TLE. At the final visit in 2021, the visual acuity values for her right and left eyes were 1.2 (1.5 × S+ 0.5D = C − 1.0DAx15°) and 0.8 (1.5× S− 0.5D), respectively. Her right and left eye IOPs were 12 and 15 mmHg, respectively.

**Figure 2:**
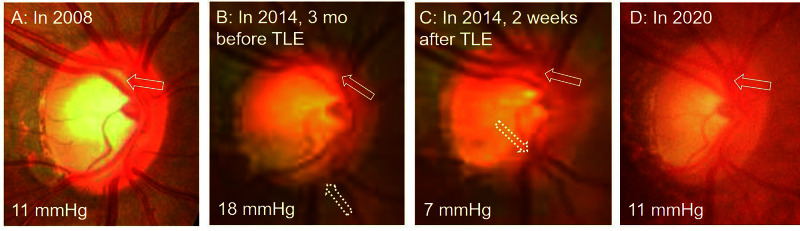
Optic disc morphological changes during 12 years of follow-up (A) Optic disc appearance three months after starting medication in 2008: the optic disc appears oval (H/V ratio = 0.78) and the C/D ratio was 0.72. (B) Optic disc appearance before TLE in 2014: compared to 2008, inferior and superior rim widths appear thinner, optic cupping appears enlarged (C/D ratio = 0.78), and optic disc is becoming circular (H/V ratio = 1.0). (C) Optic disc after two weeks of TLE: optic cupping appears to be decreasing (C/D ratio = 0.61) and the origin of the blood vessels is closer to the center. The H/V ratio was 1.0. (D) Optic disc in 2020: it has regained its oval shape (H/V ratio = 0.84) and the rim width and area are similar to those of 2008 (C/D ratio = 0.72) (A). H/V, horizontal/vertical disc diameter; C/D, vertical cup/disc diameter

In Figure 2, the morphological changes in the optic disc demonstrate structural recovery during 12 years of patient follow-up after TLE.

## Discussion

Our report shows the recoveries of optic disc shape (structure) and functionally maintained visual field during long-term follow-up after TLE. A previous analysis shows that TLEs are associated with significantly stable optic disc progression (3% vs. 10%, TLE vs. medication, respectively; P = 0.07) and more incidences of reversal of cupping (13% vs. 1%, TLE vs. medication, respectively; P < 0.001) [14]. Moreover, such reversal of cupping, i.e., displacement of the lamina cribrosa, after TLE is associated with greater IOP reduction, younger age, and smaller baseline C/D ratios [15]. Additionally, cases in which reversal cupping is large are associated with a gain of +3dB in MD [15]. These findings suggest that TLE is advantageous when patients are young and at the early stage of glaucoma. This is supported by a recent report that early visual field improvement after TLE is more likely to occur in patients with mild/moderate glaucoma, whereas the visual field of patients with severe glaucoma will tend to decline greatly over one year [16]. This is consistent with our case report: the patient was 35 years old and with moderate glaucoma (MD = −9.59) at the time of TLE. TLE is also beneficial by reducing IOP fluctuation. For instance, the diurnal fluctuation in IOP may decrease from 8.6 to 4.9 mmHg, and this effect is more pronounced in phakic eyes [17]. This observation is particularly relevant to ‘low-teens normal-tension’ type of glaucoma, because a high degree of long-term diurnal IOP fluctuation is a significant risk factor in the progression of glaucoma [18]. In our case, IOP fluctuated by approximately 8 mmHg in the three years prior to TLE, which was reduced to approximately 2 mmHg after TLE. The change in the optic disc shape (oval at baseline to circular before TLE) has been explained as the effects of IOP on the surface of the anterior lamina, which deforms the lamina posteriorly, and on the sclera, which causes an expansion of the scleral canal [19]. Therefore, decreasing IOP by TLE will induce structural changes of sclera, which is associated with changes in optic disc appearances. Furthermore, clinicians occasionally observe MD aggravation in the initial years after TLE; however, it is not possible to determine whether this phenomenon depends on the momentum of worsening glaucoma or invasion of TLE. This case suggests that it is best to observe the phenomenon patiently if the IOP control appears to be good after TLE.

Our parameter of C/D ratio, H/V ratio using optic disc photography and MD may provide subjective impressions. It is more persuasive to show objective data using optical coherence tomography (OCT) images. During the 12 years, however, the OCT instruments were replaced, and the same instrument could only be used for a few years at most. Therefore, comparing parameters such as optic disc shape, circumpapillary retinal nerve fiber layer, and macula ganglion cells measured by the same OCT during 12-year treatment was impossible. The true evaluation of structural and functional recovery after TLE should be judged by MD and same OCT parameters (no previous study done).

## Conclusions

Glaucoma treatment now requires longer time follow-up due to the long life span. Successful TLE followed by long-term rigorous IOP control can ensure structural recoveries and treat the visual field deterioration at early phase after TLE by long-term follow-up.

## References

[1] Nichani P, Popovic MM, Schlenker MB, Park J, Ahmed IIK (2020). Micro-invasive glaucoma surgery: a review of 3476 eyes. (Online ahead of print). Surv Ophthalmol.

[2] Xin C, Wang H, Wang N (2020). Minimally invasive glaucoma surgery: what do we know? Where should we go?. Transl Vis Sci Technol.

[3] Bell K, de Padua Soares Bezerra B, Mofokeng M, Montesano G, Nongpiur ME, Marti MV, Lawlor M (2021). Learning from the past: mitomycin C use in trabeculectomy and its application in bleb-forming minimally invasive glaucoma surgery. Surv Ophthalmol.

[4] Kasahara M, Shoji N (2021). Effectiveness and limitations of minimally invasive glaucoma surgery targeting Schlemm’s canal. Jpn J Ophthalmol.

[5] Feldman RM, Bell NP (2012). Complications of Glaucoma Surgery. https://global.oup.com/academic/product/complications-of-glaucoma-surgery-9780195382365?cc=us&lang=en&.

[6] Sanchez FG, Sanders DS, Moon JJ, Gardiner SK, Reynaud J, Fortune B, Mansberger SL (2020). Effect of trabeculectomy on OCT measurements of the optic nerve head neuroretinal rim tissue. Ophthalmol Glaucoma.

[7] Lee EJ, Kim TW, Weinreb RN, Kim H (2013). Reversal of lamina cribrosa displacement after intraocular pressure reduction in open-angle glaucoma. Ophthalmology.

[8] Kadziauskienė A, Jašinskienė E, Ašoklis R (2018). Long-term shape, curvature, and depth changes of the lamina cribrosa after trabeculectomy. Ophthalmology.

[9] Lee EJ, Kim TW, Weinreb RN (2013). Variation of lamina cribrosa depth following trabeculectomy. Invest Ophthalmol Vis Sci.

[10] Gietzelt C, Lemke J, Schaub F (2018). Structural reversal of disc cupping after trabeculectomy alters Bruch membrane opening-based parameters to assess neuroretinal rim. Am J Ophthalmol.

[11] Caprioli J, de Leon JM, Azarbod P (2016). Trabeculectomy can improve long-term visual function in glaucoma. Ophthalmology.

[12] Swinnen S, Stalmans I, Zeyen T (2010). Reversal of optic disc cupping with improvement of visual field and stereometric parameters after trabeculectomy in young adult patients (two case reports). Bull Soc Belge Ophtalmol.

[13] Yuen D, Buys YM (2010). Disc photography and Heidelberg retinal tomography documentation of reversal of cupping following trabeculectomy. Graefes Arch Clin Exp Ophthalmol.

[14] Parrish RK 2nd, Feuer WJ, Schiffman JC (2009). Five-year follow-up optic disc findings of the Collaborative Initial Glaucoma Treatment Study. Am J Ophthalmol.

[15] Esfandiari H, Efatizadeh A, Hassanpour K, Doozandeh A, Yaseri M, Loewen NA (2018). Factors associated with lamina cribrosa displacement after trabeculectomy measured by optical coherence tomography in advanced primary open-angle glaucoma. Graefes Arch Clin Exp Ophthalmol.

[16] Chua J, Kadziauskienė A, Wong D (2020). One year structural and functional glaucoma progression after trabeculectomy. Sci Rep.

[17] Wasielica-Poslednik J, Schmeisser J, Hoffmann EM, Weyer-Elberich V, Bell K, Lorenz K, Pfeiffer N (2017). Fluctuation of intraocular pressure in glaucoma patients before and after trabeculectomy with mitomycin C. PLoS One.

[18] Baek SU, Ha A, Kim DW, Jeoung JW, Park KH, Kim YK (2020). Risk factors for disease progression in low-teens normal-tension glaucoma. Br J Ophthalmol.

[19] Crawford Downs J, Roberts MD, Sigal IA (2011). Glaucomatous cupping of the lamina cribrosa: a review of the evidence for active progressive remodeling as a mechanism. Exp Eye Res.

